# The impact of *APOE* genotype on survival: Results of 38,537 participants from six population-based cohorts (*E2-CHARGE*)

**DOI:** 10.1371/journal.pone.0219668

**Published:** 2019-07-29

**Authors:** Frank J. Wolters, Qiong Yang, Mary L. Biggs, Johanna Jakobsdottir, Shuo Li, Daniel S. Evans, Joshua C. Bis, Tamara B. Harris, Ramachandran S. Vasan, Nuno R. Zilhao, Mohsen Ghanbari, M. Arfan Ikram, Lenore Launer, Bruce M. Psaty, Gregory J. Tranah, Alexander M. Kulminski, Vilmundur Gudnason, Sudha Seshadri, for the E2-CHARGE investigators

**Affiliations:** 1 Department of Epidemiology, Erasmus Medical Centre, Rotterdam, the Netherlands; 2 Department of Biostatistics, Boston University School of Public Health, Boston, Massachusetts, United States of America; 3 Cardiovascular Health Research Unit, Department of Medicine, University of Washington, Seattle, Washington, United States of America; 4 Department of Biostatistics, University of Washington, Seattle, Washington, United States of America; 5 Faculty of Medicine, University of Iceland, Reykavik, Iceland; 6 California Pacific Medical Center Research Institute, San Francisco, California, United States of America; 7 Laboratory of Epidemiology, Demography, and Biometry, National Institute on Aging, Bethesda, Maryland, United States of America; 8 Sections of Preventive Medicine and Epidemiology, and Cardiology, Department of Medicine, Boston University School of Medicine, Boston, Massachusetts, United States of America; 9 Icelandic Heart Association, Kopavogur, Iceland; 10 Departments of Epidemiology and Health Services, University of Washington, Seattle, Washington, United States of America; 11 Kaiser Permanente Washington Health Research Institute, Seattle, Washington, United States of America; 12 Biodemography of Aging Research Unit, Social Science Research Institute, Duke University, Durham, North Carolina, United States of America; 13 Department of Neurology, Boston University School of Medicine, Boston, Massachusetts, United States of America; Nathan S Kline Institute, UNITED STATES

## Abstract

**Background:**

Apolipoprotein E is a glycoprotein best known as a mediator and regulator of lipid transport and uptake. The *APOE-*ε4 allele has long been associated with increased risks of Alzheimer's disease and mortality, but the effect of the less prevalent *APOE-*ε2 allele on diseases in the elderly and survival remains elusive.

**Methods:**

We aggregated data of 38,537 individuals of European ancestry (mean age 65.5 years; 55.6% women) from six population-based cohort studies (Rotterdam Study, AGES-Reykjavik Study, Cardiovascular Health Study, Health-ABC Study, and the family-based Framingham Heart Study and Long Life Family Study) to determine the association of *APOE*, and in particular *APOE-*ε2, with survival in the population.

**Results:**

During a mean follow-up of 11.7 years, 17,021 individuals died. Compared with homozygous *APOE-*ε3 carriers, *APOE-*ε2 carriers were at lower risk of death (hazard ratio,95% confidence interval: 0.94,0.90–0.99; *P* = 1.1*10^−2^), whereas *APOE-*ε4 carriers were at increased risk of death (HR 1.17,1.12–1.21; *P* = 2.8*10^−16^). *APOE* was associated with mortality risk in a dose-dependent manner, with risk estimates lowest for homozygous *APOE-*ε2 (HR 0.89,0.74–1.08), and highest for homozygous *APOE-*ε4 (HR 1.52,1.37–1.70). After censoring for dementia, effect estimates remained similar for *APOE-*ε2 (HR 0.95,0.90–1.01), but attenuated for *APOE-*ε4 (HR 1.07,1.01–1.12). Results were broadly similar across cohorts, and did not differ by age or sex. *APOE* genotype was associated with baseline lipid fractions (e.g. mean difference(95%CI) in LDL(mg/dL) for ε2 versus ε33: -17.1(-18.1–16.0), and ε4 versus ε33: +5.7(4.8;6.5)), but the association between *APOE* and mortality was unaltered after adjustment for baseline LDL or cardiovascular disease. Given the European ancestry of the study population, results may not apply to other ethnicities.

**Conclusion:**

Compared with *APOE*-ε3, *APOE-*ε2 is associated with prolonged survival, whereas mortality risk is increased for *APOE-*ε4 carriers. Further collaborative efforts are needed to unravel the role of *APOE* and in particular *APOE-*ε2 in health and disease.

## Introduction

Apolipoprotein E is a glycoprotein best known as a mediator and regulator of lipid transport and uptake, but also has several additional physiological and pathological roles.[1] The *APOE* gene, on chromosome 19, contains four exons and codes for a 317 amino acid polypeptide that gives rise to a 299 amino acid long mature protein (34kD).[1] There are three circulating *APOE* isoforms designated *APOE*-ε4, -ε3, and -ε2, with corresponding allele frequencies of approximately 14%, 78%, and 8%, respectively.[2] Within the central nervous system, apolipoprotein E is produced mainly by astrocytes, while in peripheral tissue, it is expressed primarily in the liver and kidneys in addition to spleen, adrenals, and fatty tissue.[3,4]

Various studies, dating back as far as 25 years ago, have shown that allelic variation at the *APOE* locus impacts survival, and alters risk of hyperlipidaemia, atherosclerosis, cardiovascular disease, and in particular dementia.[5–7] Initial attention was largely focused on the *APOE*-ε4 allele, which is associated with an adverse impact on these risk factors and outcomes, including shortened survival compared with the more common ε3 allele. More recent data suggest that the *APOE*-ε2 allele might prolong survival,[8–11] but other studies do not support such an association,[12,13] and have even implicated the ε2 allele as a detrimental factor in cerebral small-vessel disease,[14] dysbetalipoproteinemia,[15] and aggressiveness of certain cancer.[16] A better understanding of the benefits and risks associated with *APOE-*ε2 carrier status, above and beyond the absence of the ε4 allele, could lead to novel preventive and treatment options for a wide variety of conditions to promote healthy aging and longevity. Yet, studies of the ε2 allele have been hampered by its low allele frequency, which results in only 1% of the population being homozygous ε2 carriers. Larger studies are therefore warranted, requiring collaborative efforts to design well-powered studies to address these questions.

We aggregated data from six large cohorts, and aimed to determine the impact of the *APOE*-ε2 allele on survival in the general population. In addition, we studied potential vascular or lipid-mediated mechanisms that might account for this association.

## Materials and methods

### Study population

This study population consisted of participants of European ancestry from six population-based cohort studies: the Framingham Heart Study (FHS), the AGES-Reykjavik Study (AGES), the Rotterdam Study (RS), the Cardiovascular Health Study (CHS), the Long Life Family Studies (LLFS), and the Health, Aging, and Body Composition study (HABC), of which FHS and LLFS are family-based cohorts. Details of the design and characteristics of participating studies have been described previously,[17–23] and are summarised below. All studies were approved by the relevant institutional review boards, and written informed consent was obtained from all participants. The current study was approved by the Boston University Medical Center institutional review board.

The **Framingham Heart Study (FHS)** was initiated to study determinants of cardiovascular disease. The original cohort was recruited in 1948 and the offspring of the Original cohort participants and offspring spouses were enrolled in 1971.[17,18] DNA was obtained for genetic studies in the 1990s from surviving Original cohort and Offspring participants. Year 1990 is considered the baseline exam for these analyses. All participants remain under continuous surveillance and deaths that occurred through 31^st^ December 2013 were included in the present analyses.

The **Age, Gene/Environment Susceptibility -Reykjavik Study (AGES)** was initiated to examine potential genetic susceptibility and gene/environment interaction.[19] Between 2002 and 2006, baseline exams were conducted in survivors from the Reykjavik Study. Follow-up information was complete till 31^st^ December 2015 via linkage to electronic medical records and vital status registry.

Between 1990 and 1993 all inhabitants of the Ommoord district in Rotterdam, The Netherlands, aged ≥55 years were invited to participate in the **Rotterdam study (RS).**[20] The cohort was subsequently expanded with inhabitants who moved into the area or reached eligible age in 2000 (≥55 years) and 2005 (≥45 years). Participants were interviewed at home and examined at the study centre every 4 years. Continuous surveillance of general practitioners' records, hospital records, and death certificates were used for identification of deaths and health events through 1^st^ January 2015.

The **Cardiovascular Health Study (CHS)** is a prospective population-based cohort study of cardiovascular disease and mortality in >65 year old Medicare-eligible adults living in four United States communities.[21] Recruitment of the initial cohort was completed in 1990 and 3,267 participants fulfilled the inclusion criteria of this study and had genotyping information available. Only European or European Americans, who consented to the use of their genetic data, were included in the present analyses. Major incident health events and deaths were identified through several methods, including 1) questionnaires completed by participants at each semi-annual contact during follow-up; 2) reports by family members; and 3) periodic searches of the Medicare Utilization database, the National Death Index, and local newspaper obituaries. Follow-up for the data used in this analysis was complete till June 30, 2014.

The **Long Life Family Study (LLFS)** enrolled families enriched for longevity via 4 field centers (Boston, New York, and Pittsburgh in the USA, and Denmark) between 2006 and 2009.[22] The recruitment protocol used the Family Longevity Selection Score (FLoSS) to identify family enriched of exceptional longevity, and enrolled 583 families with a FLoSS >7 consisting of 1493 probands, their siblings and 192 spouses in the older generation, and 2437 offspring and 809 of their spouses. Information collected on onsets of diseases was assessed retrospectively at baseline from self-reports and prospectively during in-person visit (home or clinic), self-administration, or telephone interview through 2015. Death was assessed annually by interview of proxies or from nationwide survival and health register (Denmark) through 2015.

The **Health, Aging, and Body Composition study (HABC)** is a prospective cohort study of 3,075 community-dwelling black and white men and women living in Memphis, TN, or Pittsburgh, PA, and aged 70–79 years at recruitment in 1996–1997.[23] Participants were a random sample of Medicare-eligible elders within designated zip code areas. The present analyses include participants of self-designated European ancestry, who consented to the use of their genetic data. After baseline examination, participants were re-examined annually, and surveilled through phone contacts every 6 months to identify major health events and document functional status between clinic visits. In addition, the study collects and abstracts medical records of all hospitalizations (≥24 hours) and adjudicates the occurrence of targeted health events including all deaths. Dates and causes of death were obtained from death certificates until September 2014. A Health ABC Committee representing all the study units adjudicated causes of death based on the review of medical records, proxy information and autopsy report (when performed).

### *APOE* genotyping

*APOE* genotype was determined directly (i.e. not using genetic imputations) in all cohorts. Methods that were used include polymerase chain reaction on coded DNA samples (RS original cohort, FHS 1^st^ and 2^nd^ generation, CHS, AGES, Health ABC) and bi-allelic Tacqman assays (rs7412 and rs429358) (RS expansion cohorts, FHS 3^rd^ generation, LLFS).

### Other measurements

Fasting serum total cholesterol, high-density lipoprotein (HDL), and triglycerides were measured at baseline. Low-density lipoprotein (LDL) was computed from total cholesterol, HDL and triglycerides, using Friedewald’s formula.[24] Use of lipid-lowering medication was assessed at baseline by interview. Prevalence of heart disease (including myocardial infarction, angina (or coronary revascularisation for the Rotterdam sample), heart failure, and cerebrovascular disease (including stroke and transient ischemic attack) was ascertained by interview, and confirmed by medical records and/or electrocardiography.

### Statistical analysis

For each cohort separately, we used Cox proportional hazard models (with robust variance for family cohorts) to determine the association between *APOE* genotype and death, while adjusting for age, sex, and center of ascertainment (if applicable). Analyses included a comparison of each of the *APOE* genotypes to ε3:ε3, as well as a comparison of ε2 carriers (ε2:ε2 or ε2:ε3) versus ε3 homozygotes, and ε4 carriers (ε3:ε4 or ε4:ε4) versus ε3 homozygotes. For appropriateness of comparison, heterozygote ε2:ε4 carriers were excluded from the latter comparisons. In additional analyses, we investigated whether results were mediated by dementia, by excluding all participants with dementia at baseline and censoring at time of incident dementia diagnosis (upon reviewer’s request). We also explored potential interaction of *APOE* with age and sex, by testing for multiplicative interaction in the Cox model. To further assess effect modification by age, we performed a sensitivity analysis among participants aged <80 years, while censoring at age 80 (upon reviewer’s request). Additionally, we adjusted these models for ethnicity, educational attainment, and smoking (upon reviewer’s request). In studies for which information on traumatic injury was available (all but Framingham), only a fraction of deaths (328/14848 = 2.2%) was attributable to trauma (upon reviewer’s request).

Next, triglyceride levels were log-transformed to obtain a roughly normal distribution of data. We then determined per cohort differences in cholesterol, HDL, triglycerides, and LDL across *APOE* genotypes, using linear regression (mixed effects model for family cohorts), adjusting for age, sex, ascertainment center, and use of lipid-lowering medication. In two cohorts (FHS and RS), we assessed the additional variance explained by *APOE* genotype. We then repeated the survival analyses with additional adjustment for measured lipid fractions, and prevalent cardiovascular disease, as well as after adjustment for ethnicity, educational attainment, and smoking.

We used inverse variance weighted fixed and random effects models to pool hazard ratios and mean differences from separate cohorts. We formally assessed for heterogeneity between studies, determining the share of variation across studies that was due to heterogeneity rather than chance (Higgins’ I^2^ statistic).[25] In case of substantial heterogeneity (>40%), we report results of random rather than fixed effects meta-analysis.

Analyses were done using IBM SPSS Statistics version 23.0 (IBM Corp, Armonk, NY, USA) or R statistical software version 3.1.1 (‘survival’ and ‘meta’ packages). Alpha level (type 1 error) was set at 0.05.

### Role of the funding source

None of the funders were involved in study design, data collection and analysis, preparation of the manuscript, or the decision to submit for publication.

## Results

A total of 38,537 participants were included from the 6 cohort studies. Baseline characteristics of the entire sample as well as per cohort are presented in Table 1. The allele frequency of the *APOE-*ε2, ε3, and ε4 alleles was 7.9%, 78.6%, and 13.5%, respectively. Observations lay within Hardy-Weinberg equilibrium.

**Table 1:** Baseline characteristics.

	Overall sample*	AGES	CHS	FHS	HABC	LLFS	RS
Sample size	38537	5740	4397	9304	1712	4630	12754
Age, years	65.5	77.0 (±5.9)	72.8 (±5.6)	51.2 (±15.2)	73.8 (±2.9)	70.3 (±15.8)	65.4 (±10.0)
Male sex	17091 (44.4%)	2429 (42.3%)	1904 (43.3%)	4242 (45.6%)	939 (52.3%)	2211 (47.5%)	5385 (42.2%)
Current smoking	5639 (14.6%)	679 (12.2%)	489 (11.1%)	1424 (16.3%)	111 (6.5%)	313 (7.3%)	2623 (21.2%)
Hypertension	19628 (50.9%)	4618 (81.1%)	2455 (55.9%)	2711 (31.0%)	671 (39.1%)	2252 (48.4%)	6921 (55.1%)
Body-mass index	26.9	27.0 (±4.5)	26.3 (±4.4)	27.1 (±5.2)	26.5 (±4.1)	27.1 (±4.9)	26.9 (±4.1)
Diabetes	3268 (8.5%)	740 (12.9%)	629 (14.4%)	469 (5.4%)	194 (11.3%)	180 (4.3%)	1056 (8.6%)
Total cholesterol, mg/dL	216.3	217.5 (±44.8)	211.8 (±39.2)	199.0 (±37.3)	201.5 (±37.6)	199.7 (±42.2)	238.0 (±47.9)
High-density lipoprotein, mg/dL	54.7	61.3 (±17.3)	53.7 (±15.8)	51.7 (±15.8)	51.9 (±16.3)	58.8 (±17.3)	53.1 (±15.2)
Triglycerides, mg/dL	129.3	108.6 (±60.7)	143.3 (±78.1)	130.0 (±112.1)	152.6 (±87.6)	113.4 (±72.1)	135.9 (±75.1)
Triglycerides (ln transformed)	4.73	4.57 (±0.46)	4.86 (±0.43)	4.69 (±0.56)	4.90 (±0.48)	4.59 (±0.51)	4.80 (±0.45)
Low-density lipoprotein, mg/dL	129.3	134.8 (±40.1)	130.3 (±35.6)	118.6 (±33.0)	119.8 (±33.2)	118.6 (±35.8)	139.4 (±35.9)
Lipid lowering medication	6362 (16.5%)	1249 (21.8%)	230 (5.2%)	606 (6.9%)	880 (51.4%)	2169 (43.4%)	1228 (9.7%)
*APOE* genotype							
ε3/ε3	23813 (61.9%)	3558 (62.0%)	2747 (62.5%)	6015 (64.6%)	1082 (63.2%)	3031 (65.1%)	7434 (58.3%)
ε2/ε2	239 (0.6%)	30 (0.5%)	28 (0.6%)	47 (0.5%)	13 (0.8%)	33 (0.7%)	89 (0.7%)
ε2/ε3	4721 (12.3%)	518 (9.0%)	560 (12.7%)	1140 (12.3%)	212 (12.4%)	695 (14.9%)	1605 (12.6%)
ε2/ε4	873 (2.3%)	115 (2.0%)	104 (2.4%)	183 (2.0%)	28 (1.6%)	87 (1.9%)	357 (2.8%)
ε3/ε4	8129 (21.1%)	1397 (24.3%)	904 (20.6%)	1764 (19.0%)	353 (20.6%)	762 (16.4%)	2965 (23.2%)
ε4/ε4	706 (1.8%)	122 (2.1%)	54 (1.2%)	155 (1.7%)	24 (1.4%)	48 (1.0%)	304 (2.4%)

N = sample size; *APOE* = apolipoprotein E; Values are depicted as mean ±SD for continuous variables, and absolute numbers (%) for categorical variables.

*derived from summary statistics

**Fig 1:**
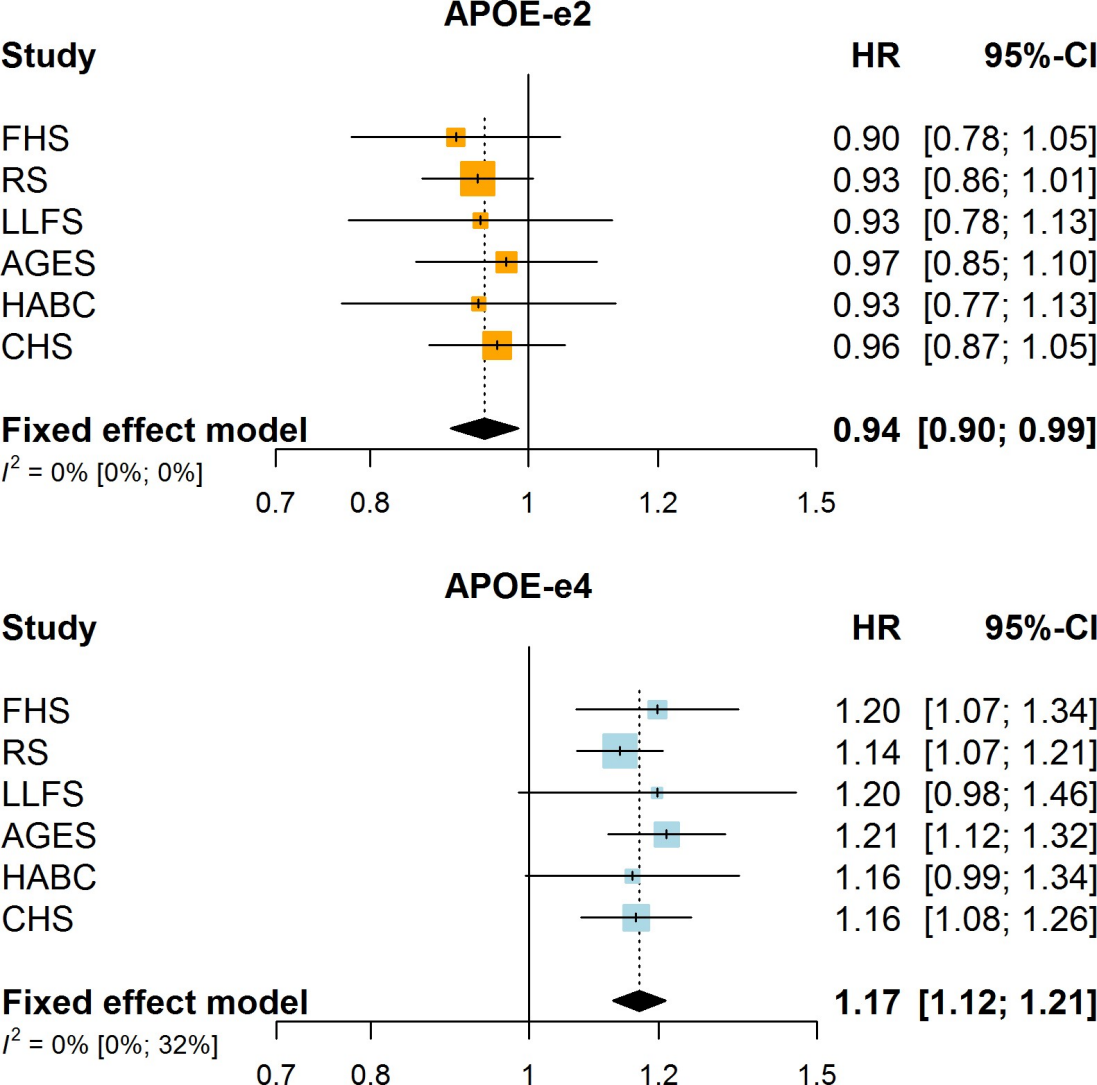
Association of *APOE*-ε2 and *APOE*-ε4 carrier status with mortality per cohort, and meta-analysis.

**Fig 2:**
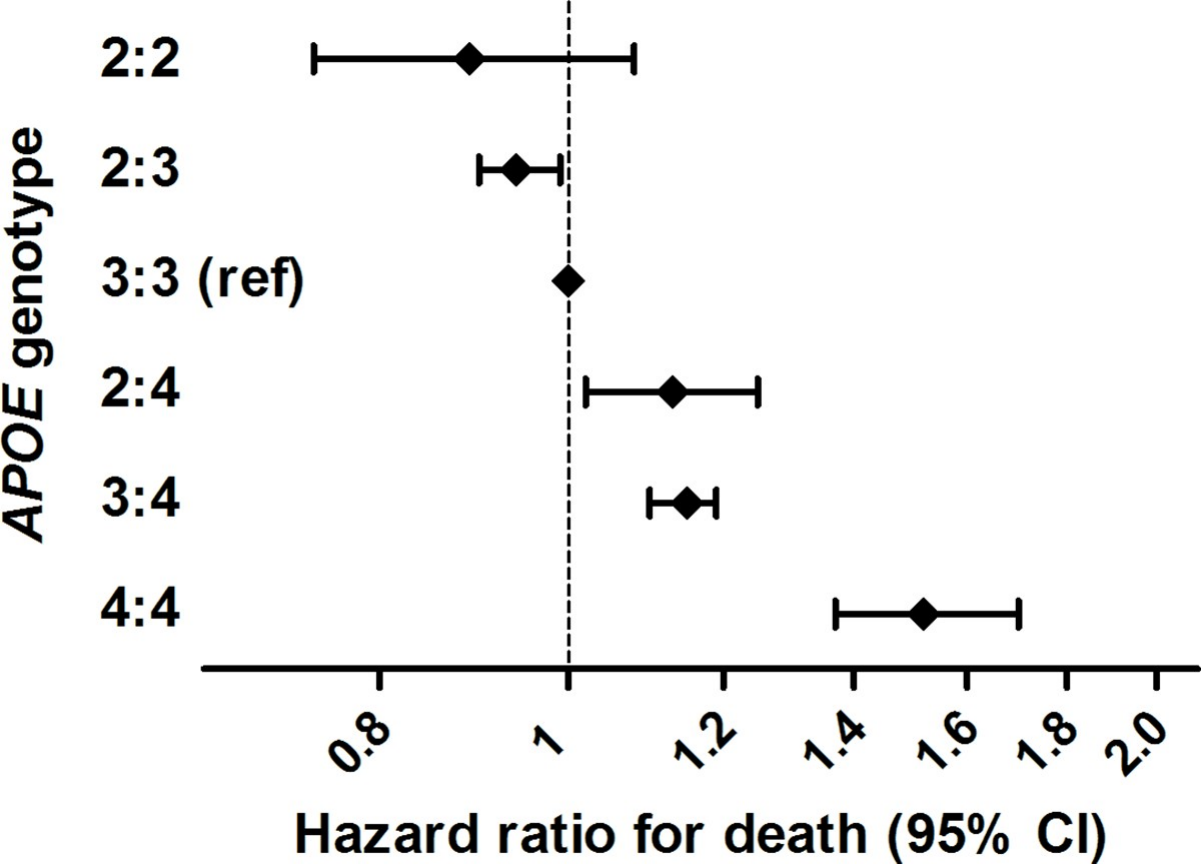
Meta-analysed effect estimates of the associations between *APOE* genotypes and mortality.

During 429,708 person years of follow-up (mean 11.7 years), 17,021 participants died. Carrying one or two copies of the ε2 allele was significantly associated with reduced mortality risk (hazard ratio (HR), 95% confidence interval: 0.94, 0.90–0.99, *P* = 1.1*10^−2^; Fig 1), whereas *APOE*-ε4 carriers were at increased risk of death (HR 1.17, 1.12–1.21, *P* = 2.8*10^−16^; Fig 1). *APOE* genotype was associated with survival in a dose-dependent manner, such that mortality risk was lowest for homozygous ε2 carriers, and highest for homozygous ε4 carriers (Fig 2). Risk for individuals with the ε2:ε4 genotype was most comparable to their ε3:ε4 rather than their ε2:ε3 counterparts.

Of all deaths, 5790 (34.0%) were attributed to cardiovascular causes, and 3922 (23.0%) to cancer. After exclusion of patients with dementia at baseline, and censoring in the main analysis at time of incident dementia diagnosis, effect estimates for mortality remained similar for *APOE-*ε2 (HR 0.95, 0.90–1.01), but attenuated for *APOE-*ε4 (HR 1.07, 1.01–1.12) (S1 Table). All associations were broadly similar across cohorts (Fig 1; for a numeral depiction see S1 Table), and there was no evidence of interaction with age at study entry (*P*-interaction for ε2 = 0.96, and for ε4 = 0.18) or sex (*P* = 0.63 and *P* = 0.49, respectively), also witnessed by similar effect estimates when restricting analyses to participants under the age of 80 (S2 Table). These analyses were also robust to concurrent adjustment for ethnicity, educational attainment, and smoking (S2 Table).

**Fig 3:**
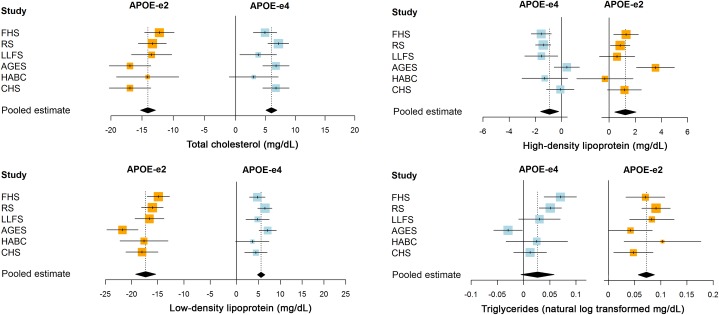
Mean differences in lipid fractions of *APOE*-ε2 (orange) and *APOE*-ε4 (light blue) compared to homozygous ε3 carriers, per cohort and meta-analysis.

**Fig 4:**
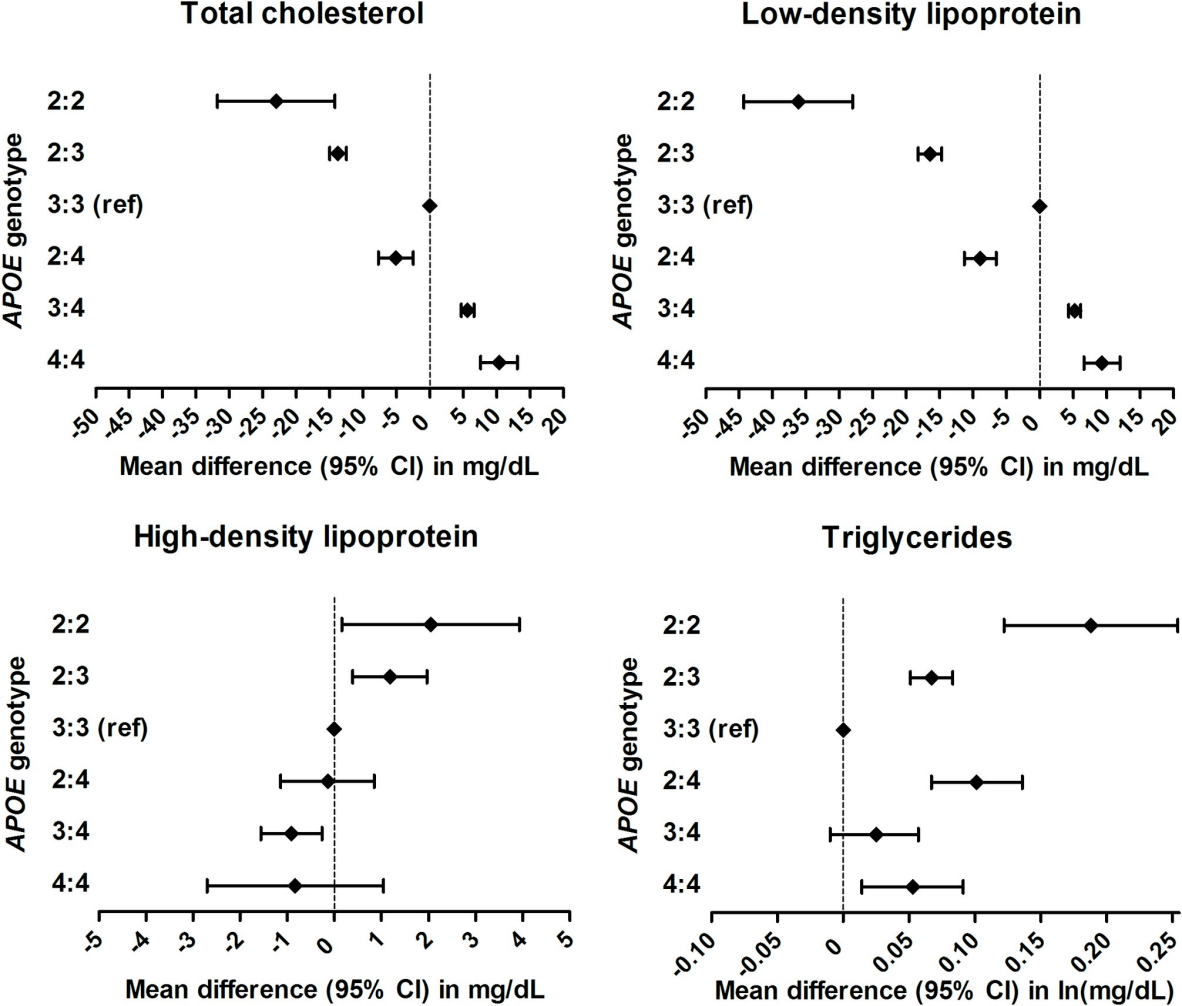
Meta-analysed effect estimates of the associations between separate *APOE* genotypes and lipid fractions.

*APOE* genotype was associated with all measured lipid fractions, generally in a dose-dependent manner (Figs 3 and 4; for full results per cohort, please see S3 Table). Compared with homozygous ε3 carriers, levels of total cholesterol, LDL, and HDL were lower in ε2 and higher in ε4 carriers, whereas both ε2 and ε4 carriers had higher levels of triglycerides. The ε2 allele was therewith associated with greater absolute changes in lipid levels than the ε4 allele. Accordingly, levels in those with ε2:ε4 genotype were generally more consistent with ε2 rather than ε4 carrier status (Fig 4). These associations were similar after additional adjustment for ethnicity, smoking, and educational attainment, except for attenuation of the relation between ε2 and HDL levels (S4 Table). Comparing ε2 and ε4 carriers with ε3 homozygotes, standardised mean differences in LDL were larger than differences in triglycerides and HDL (S5 Table). *APOE* genotype explained 1.6–3.2% of variance in total cholesterol, 3.9–5.5% for LDL, 0.3–0.4% for HDL, and 0.8–0.9% for triglycerides.

Effect estimates of *APOE* carrier status for mortality risk were not attenuated by adjustment for LDL (pooled estimates, 95%CI, for ε2 carriers: 0.91, 0.86–0.96; and for ε4 carriers: HR 1.19, 1.14–1.24). Similarly, adjustment for prevalent cardiovascular disease did not materially change risk estimates of mortality (pooled estimates, 95%CI, for ε2 carriers: 0.95, 0.90–1.00; and for ε4 carriers: HR 1.16, 1.12–1.21).

## Discussion

*E2-CHARGE* is the largest collaboration of cohort studies to date to determine the impact of *APOE* and, in particular, the *APOE-*ε2 allele, and aggregates data of 38,537 individuals from 6 population-based cohort studies. In this first analysis of the data, we found that the *APOE-*ε2 allele is associated with prolonged survival, whereas *APOE-*ε4 is associated with increased mortality. Adjustment for the prevalence of cardiovascular disease or measured lipid fractions had trivial effects on these estimates.

Since the first implication of *APOE* in longevity,[26] several genome-wide association and candidate gene studies have aimed to confirm a role of *APOE* in survival. While most of these studies indeed show that *APOE* allele frequencies shift with age,[27] or confirm that *APOE* is associated with longevity as study endpoint, they did not reach genome wide significance,[28–30] or were unable to distinguish effects attributable to variation at the *APOE* locus from that at the *TOMM40* or *APOC1* loci.[31,32] Several cohort studies have examined the association of *APOE* genotype with survival and longevity, but with contrasting findings. Two Scandinavian studies reported hazardous effects of the ε4 allele,[8,12] while one of the two studies also found a protective effect of the ε2 allele.[8] These findings are supported by a lower prevalence of ε4 and a higher prevalence of ε2 in offspring from long-lived families compared to spouse controls,[9] as well as in the very elderly compared to middle-aged populations.[10,11] Nevertheless, neither ε2 nor ε4 were prospectively associated with survival in a very elderly U.S. population, the 90+ Study.[13] We found dose-dependent associations of *APOE* genotype with survival, which were consistent across participating cohorts. Our pooled effect estimates suggest prior studies were likely underpowered to detect these differences, in particular for the ε2 allele. Survival and selection bias at older ages, whereby ε4 carriers die prior to entry or are less likely to be enrolled due to poor health, may have led to underestimation of hazardous effects of the ε4 allele in others.

After accounting for dementia in our study, associations with mortality attenuated for *APOE-*ε4, but were virtually unaltered for *APOE-*ε2. This suggests that a vast part of excess risk for *APOE-*ε4 is via its effect on dementia pathology, but that other mechanisms also play a role in carriers of the *APOE-*ε2 and to a lesser extent *APOE-*ε4 allele. Mounting evidence indeed suggests pleiotropic effects of *APOE* on various organ systems. In addition to the well-established link with dementia, more recent clinical and population studies have linked *APOE* gene variation to atherosclerosis,[33] cerebral amyloid angiopathy,[34] stroke,[33] lung disease,[35] multiple sclerosis,[36] and neoplasia.[37] Preclinical studies have put forward intriguing hypotheses of molecular pathways, relating to cerebrovascular function,[38,39] neuronal growth regulation,[40] inflammation,[3] functions as a protein chaperone,[41] type III hyperlipoproteinemia,[15,42] prostate tumor aggressiveness,[16] and epigenetic regulation of the transcriptional pattern at the *APOE* locus by DNA methylation.[43] The associations we found of *APOE* genotype with lipid fractions align well with those in a prior European study.[33] We investigated circulating lipid fraction concentrations as a potential underlying mechanism of prolonged survival in *APOE-*ε2 carriers, but found no evidence of mediation. In addition, estimates changed only minimally after taking into account clinically manifest vascular disease at baseline. Although this may in part reflect limitations of single measurements of lipid fractions, it suggests other (potentially age- and sex-specific) mechanisms could be involved.[44]

Certain limitations should be taken into account. First, although we only determined *APOE* genotype directly rather than by imputation, we did not investigate other genetic variants that might modify the effect of *APOE* through epistatic interactions. Second, (impact of) serum lipid levels on health and disease may differ over time, which is not captured by one-time measurement at study baseline, and may cause underestimation of any mediation. Third, Friedewald’s formula for computation of LDL levels assumes that all triglycerides are carried on VLDL, and that the triglyceride-to-cholesterol-ratio of VLDL is constant at 5:1, which may not apply in all individuals. Fourth, although the mean age of participants at study entry was only 65 years, we cannot rule out attenuation of effect estimates, in particular for ε4 carriers, due to selection bias at older ages. Fifth, albeit the largest study of *APOE-*ε2 in relation to mortality to date, precision may still be lacking with respect to separate genotypes to fully reveal a dose-effect response. Finally, the study population was entirely of European ancestry, and findings may not be applicable to other ethnicities.

In conclusion, *E2-CHARGE* brings together data from several population-based studies worldwide. In this paper, we describe the details of each population and the first analysis of the data. We find that *APOE-*ε2 prolongs survival in the general population of European descent, which appears only in part explained by commonly determined lipid fractions, or prevalent vascular disease. Further studies are needed to determine the role of *APOE-*ε2 in vascular, as well as other types of disease, above and beyond the absence of the *APOE-*ε4 allele. Various other population studies have collected or are collecting data on *APOE* genotype and disease outcomes, and inclusion of these data–in particular from ethnically diverse populations–may aid in elucidating the role of *APOE-*ε2 in health and disease.

## Supporting information

S1 Supporting Information Funding information per cohort. (DOCX)

S2 Supporting Information Data availability statement. (DOCX)

S1 Table Individual study results of associations between different *APOE* genotypes and mortality. (DOCX)

S2 Table Associations between *APOE* genotypes and mortality in participants <80 years (including censoring at age 80), with additional adjustment for ethnicity, smoking, and educational attainment. (DOCX)

S3 Table Individual study results of associations between different *APOE* genotypes and lipid fractions. (DOCX)

S4 Table Associations between different *APOE* genotypes and lipid fractions after additional adjustment for ethnicity, smoking, and educational attainment. (DOCX)

S5 Table Meta-analysis of standardised effect estimates for the association between different *APOE* genotypes and lipid fractions. (DOCX)
