# Supplementary material for: The impact of APOE genotype on survival: Results of 38,537 participants from six population-based cohorts (E2-CHARGE)
Source: PLoS One. 2019 Jul 29;14(7):e0219668. doi: 10.1371/journal.pone.0219668 (PMC6663005; doi:10.1371/journal.pone.0219668)
Supplement: S1 Supporting Information — (DOCX) [file pone.0219668.s001.docx]

**S1 Supporting Information – Sources of funding for individual cohorts**

**Long Life Family Study (LLFS).** Sponsored by the National Institute on Aging (NIA cooperative agreements U01-AG023712, U01-AG23744, U01-AG023746, U01-AG023749, and U01-AG023755). The analyses were supported by the NIA grants U01-AG023712, P01-AG043352, and R01-AG047310.

**Health ABC.** This work was supported by National Institute on Aging (NIA) Contracts N01-AG-6-2101; N01-AG-6-2103; N01-AG-6-2106; NIA grant R01-AG028050, and National Institute of Nursing Research (NINR) grant R01-NR012459. This research was supported in part by the Intramural Research Program on the National Institutes of Health (NIH), National Institute on Aging.

**Age, Gene/Environment Susceptibility (AGES).** This study is supported by National Institute of Aging contracts (N01-AG-12100 and HHSN271201200022C) with contributions from the National Eye Institute, National Institute on Deafness and Other Communication Disorders, and the National Heart, Lung and Blood Institute, the National Institute of Aging Intramural Research Program, Hjartavernd (the Icelandic Heart Association), and the Althingi (the Icelandic Parliament).

**Cardiovascular Health Study (CHS).** This CHS research was supported by NHLBI contracts HHSN268201200036C, HHSN268200800007C, N01HC55222, N01HC85079, N01HC85080, N01HC85081, N01HC85082, N01HC85083, N01HC85086; and NHLBI grants U01HL080295, R01HL087652, R01HL105756, R01HL103612, R01HL120393, and R01HL130114 with additional contribution from the National Institute of Neurological Disorders and Stroke (NINDS). Additional support was provided through R01AG023629 from the National Institute on Aging (NIA). A full list of principal CHS investigators and institutions can be found at CHS-NHLBI.org. The provision of genotyping data was supported in part by the National Center for Advancing Translational Sciences, CTSI grant UL1TR000124, and the National Institute of Diabetes and Digestive and Kidney Disease Diabetes Research Center (DRC) grant DK063491 to the Southern California Diabetes Endocrinology Research Center. The content is solely the responsibility of the authors and does not necessarily represent the official views of the National Institutes of Health.

**Framingham Heart Study (FHS)**. This work was supported by the National Heart, Lung, and Blood Institute’s Framingham Heart Study (contracts N01-HC-25195 and HHSN268201500001I). This study was also supported by grants from the National Institute on Aging: (AG054076, U01-AG049505, and AG008122 (S. Seshadri)). S. Seshadri and A. Beiser were also supported by additional grants from the National Institute on Aging (R01AG049607, AG033193, AG033040) and the National Institute of Neurological Disorders and Stroke (R01-NS017950).

**The Rotterdam Study.** This study is supported by the Erasmus Medical Centre and Erasmus University Rotterdam, The Netherlands Organization for Scientific Research (NWO), The Netherlands Organization for Health Research and Development (ZonMW), the Research Institute for Diseases in the Elderly (RIDE), The Netherlands Genomics Initiative, the Ministry of Education, Culture and Science, the Ministry of Health, Welfare and Sports, the European Commission (DG XII), and the Municipality of Rotterdam. Further support was obtained from the Netherlands Consortium for Healthy Ageing and the Dutch Heart Foundation (2012T008). This research was further supported by funding from the European Union Seventh Framework Program (FP7/2007e2013) under grant agreement no. 601055, VPH-Dare@IT (FP7-ICT-2011-9e601055); and funding from the European Union's Horizon 2020 research and innovation program under grant agreement no. 667375 (Co-STREAM) and under grant agreement no. 678543 (European Research Council (ERC) funded project: ORACLE).
