# Supplementary material for: The impact of APOE genotype on survival: Results of 38,537 participants from six population-based cohorts (E2-CHARGE)
Source: PLoS One. 2019 Jul 29;14(7):e0219668. doi: 10.1371/journal.pone.0219668 (PMC6663005; doi:10.1371/journal.pone.0219668)
Supplement: S2 Supporting Information — (DOCX) [file pone.0219668.s002.docx]

**S2 Supporting Information – Data availability statement**

Data of several U.S. based cohorts are available through dbGaP. Qualified researchers can apply for authorization to access the relevant depositories via the following URLs. Detailed instruction for application is provided here: <https://www.ncbi.nlm.nih.gov/projects/gap/cgi-bin/GetPdf.cgi?document_name=GeneralAAInstructions.pdf>.

**Framingham Study**: <https://www.ncbi.nlm.nih.gov/projects/gap/cgi-bin/study.cgi?study_id=phs000007.v30.p11>

**Cardiovascular Health Study**: <https://www.ncbi.nlm.nih.gov/projects/gap/cgi-bin/study.cgi?study_id=phs000287.v6.p1>

**Long Life Family Study**: <https://www.ncbi.nlm.nih.gov/projects/gap/cgi-bin/dataset.cgi?study_id=phs000397.v1.p1&pht=2410>; for LLFS, outside investigators (with an LLFS sponsor) can apply directly to LLFS to obtain the data. Outside investigators will be able to participate in the writing of manuscripts through collaboration with a LLFS investigator. The sponsoring investigator will send a cover letter of introduction with the proposal to the committee. The sponsoring investigator will facilitate access to the study policies and procedures. The Coordinating Center will release data to an outside investigator upon approval of the proposal along with documentation of IRB approval, Data Distribution agreement, and human subject certification at the proposer's home institution. The ancillary study must be proposed with an LLFS investigator as a co-investigator and at least three LLFS investigators must be included as co-authors on these manuscripts.

**Health ABC:** genotype data via <https://www.ncbi.nlm.nih.gov/projects/gap/cgi-bin/study.cgi?study_id=phs000169.v1.p1>. Phenotype data can be applied for via <https://healthabc.nia.nih.gov/>, with detailed procedures outlined here: <https://healthabc.nia.nih.gov/analysis-proposals-publications>.

**Age, Gene/Environment Susceptibility:** data can be made available to interested researchers upon request following approval by the relevant institutional review boards. Requests can be directed to the Icelandic Heart Association ([AGES_data_request@hjarta.is](mailto:AGES_data_request@hjarta.is)). Data sharing is in accordance with the informed consent after an approval by the Icelandic National Bioethics committee and the Data Protection Authority of Iceland.

**Rotterdam Study** data can be made available to interested researchers upon request. Requests can be directed to data manager Frank J.A. van Rooij (f.vanrooij@erasmusmc.nl). We are unable to place data in a public repository due to legal and ethical restraints on the basis of the EU General Data Protection Regulation (GDPR), and its implementation in the Dutch ‘Algemene verordening gegevensbescherming’ (AVG). Sharing of individual participant data was not included in the informed consent of the study, and there is potential risk of revealing participants’ identities as it is not possible to completely anonymize the data. This is of particular concern given the sensitive personal nature of much of the data collected as part of the Rotterdam Study.
