# Supplementary material for: The impact of APOE genotype on survival: Results of 38,537 participants from six population-based cohorts (E2-CHARGE)
Source: PLoS One. 2019 Jul 29;14(7):e0219668. doi: 10.1371/journal.pone.0219668 (PMC6663005; doi:10.1371/journal.pone.0219668)
Supplement: S1 Table — (DOCX) [file pone.0219668.s003.docx]

**S1 Table** Individual study results of associations (hazard ratio, 95% confidence interval) between different *APOE* genotypes and mortality.

|  | N_mort_/N_total_ | AGES | CHS | FHS | HABC | LLFS | RS |
| --- | --- | --- | --- | --- | --- | --- | --- |
| **Overall analysis** |  |  |  |  |  |  |  |
| *APOE* genotype |  |  |  |  |  |  |  |
| ε2/ε2 | 107/239 | 0.74, 0.41-1.35 | 0.96, 0.65-1.43 | 1.20, 0.70-2.08 | 0.67, 0.30-1.50 | 1.13, 0.53-2.42 | 0.82, 0.61-1.10 |
| ε2/ε3 | 2027/4728 | 0.98, 0.86-1.12 | 0.95, 0.87-1.05 | 0.89, 0.76-1.03 | 0.96, 0.79-1.17 | 0.94, 0.78-1.13 | 0.94, 0.87-1.02 |
| ε3/ε3 | 10405/23847 | Reference | Reference | Reference | Reference | Reference | Reference |
| ε2/ε4 | 410/873 | 1.20, 0.94-1.53 | 0.98, 0.79-1.22 | 1.25, 0.86-1.82 | 1.44, 0.90-2.29 | 1.02, 0.61-1.70 | 1.15, 0.99-1.34 |
| ε3/ε4 | 3747/8143 | 1.19, 1.09-1.29 | 1.14, 1.06-1.24 | 1.15, 1.03-1.30 | 1.16, 0.99-1.36 | 1.17, 0.96-1.43 | 1.12, 1.05-1.19 |
| ε4/ε4 | 325/707 | 1.54, 1.21-1.95 | 1.59, 1.21-2.10 | 1.99, 1.49-2.64 | 1.25, 0.76-2.05 | 1.89, 0.86-4.16 | 1.38, 1.17-1.63 |
|  |  |  |  |  |  |  |  |
| **Censoring for dementia^*^** |  |  |  |  |  |  |  |
| *APOE* genotype |  |  |  |  |  |  |  |
| ε2/ε2 | 63/213 | 0.82, 0.37-1.83 | 0.61, 0.15-2.47 | 0.77, 0.31-1.93 | 0.66, 0.29-1.46 | 1.12, 0.52-2.41 | 0.86, 0.62-1.20 |
| ε2/ε3 | 1220/4231 | 1.01, 0.85-1.19 | 0.89, 0.66-1.19 | 0.96, 0.82-1.12 | 1.09, 0.89-1.33 | 0.92, 0.75-1.13 | 0.94, 0.86-1.04 |
| ε3/ε3 | 6044/21038 | Reference | Reference | Reference | Reference | Reference | Reference |
| ε2/ε4 | 217/761 | 1.04, 0.73-1.49 | 1.13, 0.60-2.12 | 1.17, 0.77-1.76 | 1.54, 0.86-2.73 | 0.98, 0.57-1.70 | 1.09, 0.91-1.31 |
| ε3/ε4 | 1878/7003 | 1.21, 1.09-1.35 | 1.08, 0.84-1.40 | 1.02, 0.88-1.17 | 1.12, 0.93-1.33 | 1.22, 0.97-1.52 | 1.00, 0.92-1.08 |
| ε4/ε4 | 128/593 | 1.24, 0.87-1.78 | 1.22, 0.50-2.96 | 1.61, 1.12-2.32 | 0.97, 0.50-1.87 | 1.34, 0.48-3.74 | 0.81, 0.61-1.07 |

* Numbers in this analysis were smaller due to exclusion of prevalent dementia and some missing data on cognitive status
