# Supplementary material for: The impact of APOE genotype on survival: Results of 38,537 participants from six population-based cohorts (E2-CHARGE)
Source: PLoS One. 2019 Jul 29;14(7):e0219668. doi: 10.1371/journal.pone.0219668 (PMC6663005; doi:10.1371/journal.pone.0219668)
Supplement: S2 Table — (DOCX) [file pone.0219668.s004.docx]

**S2 Table** Associations between *APOE* genotypes and mortality in participants <80 years (including censoring at age 80), with additional adjustment for ethnicity, smoking, and educational attainment.

|  | N_mort_/N_total_ | HR, 95% CI |
| --- | --- | --- |
| *APOE* genotype |  |  |
| ε2/ε2 | 19/172 | 0.92, 0.59-1.43 |
| ε2/ε3 | 513/3691 | 0.96, 0.87-1.08 |
| ε3/ε3 | 2741/18148 | Reference |
| ε2/ε4 | 120/676 | 1.27, 1.05-1.54 |
| ε3/ε4 | 1117/6376 | 1.11, 1.03-1.20 |
| ε4/ε4 | 131/597 | 1.50, 1.24-1.82 |
| ε2 vs ε3/ε3 | 532/3863 | 0.96, 0.87-1.05 |
| ε4 vs ε3/ε3 | 1248/6973 | 1.13, 1.05-1.21 |
