# Supplementary material for: The impact of APOE genotype on survival: Results of 38,537 participants from six population-based cohorts (E2-CHARGE)
Source: PLoS One. 2019 Jul 29;14(7):e0219668. doi: 10.1371/journal.pone.0219668 (PMC6663005; doi:10.1371/journal.pone.0219668)
Supplement: S3 Table — (DOCX) [file pone.0219668.s005.docx]

**S3 Table** Individual study results of associations (mean difference, 95% confidence interval) between different *APOE* genotypes and lipid fractions.

|  | N_total_ | AGES | CHS | FHS | HABC | LLFS | RS |
| --- | --- | --- | --- | --- | --- | --- | --- |
| **Total cholesterol** |  |  |  |  |  |  |  |
| ε2/ε2 | 235 | -32.1, -45.4;-18.8 | -45.0, -58.6;-31.4 | -30.2, -40.9;-19.5 | -7.9, -26.8;10.9 | -22.4, -36.0;-8.8 | -11.2, -20.0;-2.3 |
| ε2/ε3 | 4628 | -16.1, -19.6;-12.7 | -13.0, -16.3;-9.7 | -11.8, -14.2;-9.4 | -14.5, -19.6;-9.4 | -13.3, -16.6;-10.1 | -13.5, -15.8;-11.2 |
| ε3/ε3 | 23302 | Reference | Reference | Reference | Reference | Reference | Reference |
| ε2/ε4 | 858 | -4.3, -11.2;2.6 | -13.0, -20.2;-5.8 | -4.4, -9.8;1.1 | -5.8, -18.7;7.2 | -6.7, -15.1;1.6 | -5.7, -10.3;-1.21 |
| ε3/ε4 | 7939 | 6.1, 3.8;8.4 | 4.1, 1.4;6.9 | 4.6, 2.6;6.6 | 3.1, -1.2;7.3 | 3.7, 0.6-6.9 | 6.9, 5.1;8.7 |
| ε4/ε4 | 689 | 14.6, 7.9;21.3 | 15.8, 5.9;25.7 | 7.8, 2.0;13.7 | 3.4, -10.6;17.4 | 4.7, -6.5;15.8 | 9.5, 4.6;14.4 |
|  |  |  |  |  |  |  |  |
| **Low-density lipoprotein** |  |  |  |  |  |  |  |
| ε2/ε2 | 193 | -43.2, -55.2;-31.3 | -52.0, -65.3;-38.7 | -39.0, -49.2;-28.9 | -18.2, -37.0;0.5 | -32.6, -44.3;-20.9 | -29.0, -38.0;-20.1 |
| ε2/ε3 | 4132 | -20.6, -23.6;-17.5 | -16.0, -19.1;-12.8 | -14.2, -16.4;-12.1 | -17.6, -22.3;-12.9 | -16.1, -19.0;-13.3 | -16.0, -18.1;-13.8 |
| ε3/ε3 |  | Reference | Reference | Reference | Reference | Reference | Reference |
| ε2/ε4 | 744 | -6.9, -13.1;-0.8 | -13.0, -19.8;-6.2 | -7.6, -12.6;-2.6 | -10.1, -22.3;2.1 | -13.5, -20.7;-6.3 | -7.7, -12.0;-3.5 |
| ε3/ε4 | 7029 | 6.5, 4.4;8.6 | 4.0, 1.4;6.7 | 4.4, 2.5;6.2 | 3.3, -0.6;7.2 | 4.7, 2.0;7.4 | 6.5, 4.8;8.2 |
| ε4/ε4 | 589 | 13.4, 7.4;19.4 | 10.4, 1.0;19.7 | 7.6, 2.2;13.0 | 7.9, -4.9;20.8 | 5.1, -4.5;14.7 | 9.2, 4.6;13.8 |
|  |  |  |  |  |  |  |  |
| **High-density lipoprotein** |  |  |  |  |  |  |  |
| ε2/ε2 | 235 | 5.6, -0.3;11.4 | 0.4, -5.0;5.8 | 5.6, 1.4;9.7 | 0.1, -8.3;8.4 | 0.6, -5.0;6.2 | 0.5, -2.4;3.5 |
| ε2/ε3 | 4619 | 3.4, 1.9;4.9 | 1.2, -0.1;2.5 | 1.2, 0.2;2.1 | -0.4, -2.6;1.9 | 0.4, -0.9;1.8 | 0.9, 0.1;1.6 |
| ε3/ε3 | 23277 | Reference | Reference | Reference | Reference | Reference | Reference |
| ε2/ε4 | 856 | 1.4, -1.6;4.4 | -2.1, -4.9;0.7 | -0.4, -2.5;1.7 | -2.5, -8.2;3.2 | 0.9, -2.6;4.3 | 0.1, -1.4;1.6 |
| ε3/ε4 | 7921 | 0.4, -0.6;1.4 | -0.3, -1.4;0.8 | -1.4, -2.2;-0.6 | -1.2, -3.0;0.6 | -1.6, -2.9;-0.3 | -1.3, -2.0;-0.7 |
| ε4/ε4 | 688 | 0.1, -2.8;3.0 | 3.8, -0.2;7.7 | -3.2, -5.5;-0.9 | -2.4, -8.5;3.8 | 0.4, -4.2;4.9 | -1.9, -3.5;-0.3 |
|  |  |  |  |  |  |  |  |
| **Triglycerides*** |  |  |  |  |  |  |  |
| ε2/ε2 | 185 | 0.22, 0.06;0.38 | 0.06, -0.10;0.22 | 0.13, -0.04;0.30 | 0.23, -0.06;0.52 | 0.25, 0.07;0.43 | 0.25, 0.13;0.37 |
| ε2/ε3 | 3930 | 0.03, -0.01;0.07 | 0.05, 0.01;0.09 | 0.07, 0.03;0.10 | 0.10, 0.02;0.17 | 0.08, 0.03;0.12 | 0.08, 0.05;0.11 |
| ε3/ε3 | 20759 | Reference | Reference | Reference | Reference | Reference | Reference |
| ε2/ε4 | 722 | 0.06, -0.02;0.15 | 0.09, 0.004;.17 | 0.08, -0.01;0.17 | 0.15, -0.05;0.34 | 0.11, 0.003;0.22 | 0.12, 0.06;0.18 |
| ε3/ε4 | 6700 | -0.04, -0.06;-0.01 | 0.01, -0.02;0.04 | 0.06, 0.03;0.10 | 0.03, -0.03;0.09 | 0.03, -0.01;0.08 | 0.05; 0.03;0.07 |
| ε4/ε4 | 561 | 0.03, -0.05;0.11 | 0.02, -0.09;0.14 | 0.12, 0.03;0.22 | -0.01, -0.22;0.19 | -0.03, -0.18;0.11 | 0.07; 0.01;0.13 |

*natural log-transformed
