# Supplementary material for: The impact of APOE genotype on survival: Results of 38,537 participants from six population-based cohorts (E2-CHARGE)
Source: PLoS One. 2019 Jul 29;14(7):e0219668. doi: 10.1371/journal.pone.0219668 (PMC6663005; doi:10.1371/journal.pone.0219668)
Supplement: S4 Table — (DOCX) [file pone.0219668.s006.docx]

**S4 Table** Associations between different *APOE* genotypes and lipid fractions after additional adjustment for ethnicity, smoking, and educational attainment.

|  | **Total cholesterol** | **Low-density lipoprotein** | **High-density lipoprotein** | **Triglycerides*** |
| --- | --- | --- | --- | --- |
| ε2/ε2 | -24.6, -30.0;-19.2 | -40.1, -46.0;-34.2 | -0.13, -1.03;0.77 | 0.21, 0.13;0.28 |
| ε2/ε3 | -13.5, -14.8;-12.1 | -16.4, -17.8;-15.0 | 0.05, -0.16;0.27 | 0.06, 0.05;0.08 |
| ε3/ε3 | Reference | Reference | Reference | Reference |
| ε2/ε4 | -6.0, -8.8;-3.1 | -9.2, -12.4;-6.1 | 0.02, -0.39;0.43 | 0.09, 0.06;0.13 |
| ε3/ε4 | 5.5, 4.4;6.5 | 5.3, 4.1;6.4 | -0.18, -0.32;-0.04 | 0.03, 0.01;0.04 |
| ε4/ε4 | 10.2, 7.0;13.3 | 9.6, 6.1;13.1 | -0.02, -0.43;0.39 | 0.05, 0.01;0.09 |
| ε2 vs ε3/ε3 | -13.9, -15.2;-12.6 | -17.3, -18.7;-15.9 | 0.04, -0.17;0.25 | 0.07, 0.05;0.09 |
| ε4 vs ε3/ε3 | 5.9, 4.8;6.9 | 5.6, 4.5;6.8 | -0.17, -0.41;-0.03 | 0.03, 0.01;0.04 |

*natural log-transformed
