# Supplementary material for: The impact of APOE genotype on survival: Results of 38,537 participants from six population-based cohorts (E2-CHARGE)
Source: PLoS One. 2019 Jul 29;14(7):e0219668. doi: 10.1371/journal.pone.0219668 (PMC6663005; doi:10.1371/journal.pone.0219668)
Supplement: S5 Table — (DOCX) [file pone.0219668.s007.docx]

**S5 Table** Meta-analysis of standardised effect estimates (95% confidence interval) for the association between different *APOE* genotypes and lipid fractions.

|  | **Standardised mean difference** (95% CI) |
| --- | --- |
| **Total cholesterol** |  |
| ε2 vs ε3/ε3 | -0.32, -0.35;-0.29 |
| ε4 vs ε3/ε3 | 0.13, 0.11;0.16 |
|  |  |
| **Low-density lipoprotein** |  |
| ε2 vs ε3/ε3 | -0.48, -0.51;-0.45 |
| ε4 vs ε3/ε3 | 0.16, 0.13;0.18 |
|  |  |
| **High-density lipoprotein** |  |
| ε2 vs ε3/ε3 | 0.07, 0.05;0.10 |
| ε4 vs ε3/ε3 | -0.06, -0.09;-0.04 |
|  |  |
| **Triglycerides** |  |
| ε2 vs ε3/ε3 | 0.15, 0.12;0.18 |
| ε4 vs ε3/ε3 | 0.06, 0.04;0.09 |
